# Evidence for Phospholipids on the Surface of Human Tears

**DOI:** 10.1167/iovs.61.14.19

**Published:** 2020-12-16

**Authors:** Ben J. Glasgow

**Affiliations:** 1 Departments of Pathology and Ophthalmology, UCLA School of Medicine, Jules Stein Eye Institute, Los Angeles, California, United States

**Keywords:** human tear film, ocular surface, phospholipids, PM-IRRAS, FTIR, ellipsometry, Meibomian lipids, tear lipocalin, LCN1

## Abstract

**Purpose:**

The structure of tears has been theoretically considered three tiers with lipids at the air interface, aqueous and proteins in the subphase, and anchored mucins on the corneal epithelial surface. While many lipid and protein species have been identified in tears by mass spectrometry, the localization of the major components within the tear film structure remains speculative. The most controversial components are phospholipids. Although surface active, phospholipids have been presumed to be bound entirely to protein in the aqueous portion of tears or reside at the aqueous-lipid interface. Herein, the possibility that phospholipids are adsorbed at the air-surface interface of tears is interrogated.

**Methods:**

Polarization-modulated Fourier transform infrared reflective absorption spectroscopy (PM-IRRAS) was used to study the presence of phosphate signals at the tear surface. In order to constrain the depth of signal detection to the surface, an extreme grazing angle of incident radiation was employed. Nulling ellipsometry was used to confirm the presence of monolayers and surface thicknesses when surface active reagents were added to solutions.

**Results:**

Surface selection of PM-IRRAS was demonstrated by suppression of water and phosphate signals in buffers with monolayers of oleic acid. Phosphate signals were shown to reflect relative concentrations. Absorption peaks attributable to phospholipids were detected by PM-IRRAS on the human tear film surface and were augmented by the addition of phospholipid.

**Conclusions:**

The data provide strong evidence that phospholipids are present at the surface of tears.

The human tear film is an example of a critically important biologic film that is dynamic with the surface continually repopulated with each blink. An abnormal tear film is implicated in dry eye disease, yet the tear film structure is unknown. Based on extrapolation from simplified mockups and speculation, the tear film was originally modeled as three separate tiers composed of lipid, aqueous and proteins, and tethered mucins, respectively. However, in recent years, a number of models have been proposed to challenge the traditional three-layer concept (Fig. 1).^1^^–^^6^ A common theme in most models is that lipids are present in a layer at the air interface of the tear film. The evidence for a lipid layer is based largely on interferometric studies that show a change in the index of refraction (n) at the outer boundary.^7^ The range of n for surface active proteins spans 1.35–1.6^8^ and encompasses the values for lipids, n ≈ 1.47–9.^9^^,^^10^ Both lipids and proteins differ in refractive index from aqueous (n ≈ 1.33), and the presence of either on the surface could account for the refractive change observed. Sparse chemical evidence exists to verify specific components in the presumed lipid surface layer. A recent ellipsometric study of tears fit the optical constant of n = 1.49 at a wavelength of 658 nm,^11^ matching that published for Meibomian lipids.^12^ However, studies to identify the specific lipids that reside on the surface of tears are lacking. The composition of the major lipids in tears by mass spectrometry is shown in Table 1. Of the major lipid components, several are surface active, including fatty acids and phospholipids. However, wax esters and cholesteryl esters comprise almost 80% of the tear lipids. Phospholipids are perhaps the most controversial component of the tear film. The putative sources of phospholipids in meibum and tears are cell membranes. Holocrine secretion of Meibomian glandular cells includes cell membranes.

**Figure 1.**
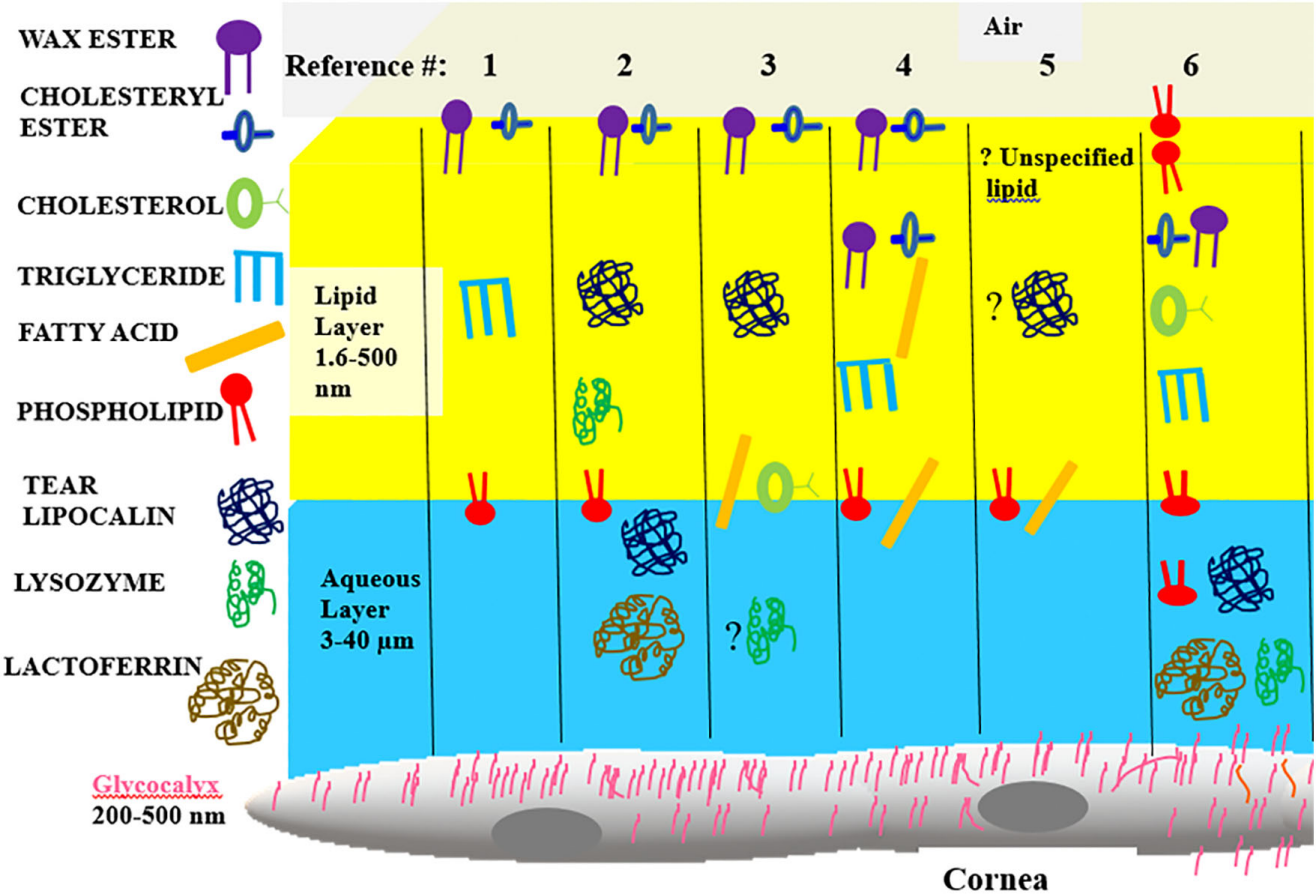
Cartoon of six published tear film models with molecular components. Key shown at *left*. Relative polarity of lipid components is shown increasing from *blue* to *red*. Published figures from the references as indicated were adapted to make symbols uniform and compare various theoretical location of components. The compartments in the cartoon are not to scale.

**Table 1.** Major Lipid and Protein Components of Tears

Component	µM ± SD	Mole %
Wax ester^13^^,^^14^	76 ± 5	35.8
Cholesteryl ester^13^^,^^14^	65 ± 64	42.7
Phospholipids^15^^–^^18^	34 ± 18	8.2
Cholesterol^15^^–^^17^	15 ± 2.4	12.9
Fatty acid^13^^,^^15^^,^^19^	Unknown	15.4
Triacylglycerol^13^^–^^18^	8 ± 3	2.3
Lysozyme^20^	162 ± 40	
Tear lipocalin^20^^–^^24^	108 ± 44	
Lactoferrin^20^	27 ± 8	
Lipophilin^25^	∼2.7	

Meibum is continually spread on the ocular surface with each blink of the eyelids. Ocular surface cells shed into the tear film are also a potential source of phospholipids. Some studies did not detect phospholipids in tears by electrospray mass spectrometry.^26^ Similarly, others did not find phospholipids in meibum with atmospheric pressure chemical ionization mass spectrometry.^27^^,^^28^ In contrast, several groups identified phospholipid species in tears and/or meibum.^29^^–^^36^ Particularly relevant to the current study, Borchman et al.^36^ found infrared bands at 1272, 1067, and 1043 cm^–1^ in extracts from tears. The bands were assigned to asymmetric and symmetric stretches of PO_2_^–^ groups and a diester stretch from C-O-P-O-C, respectively. Interestingly, these bands were not detected in meibum in the study. Phospholipids of tears have been extracted from the purified protein fraction containing tear lipocalin, also called lipocalin 1 or LCN1.^32^^,^^33^ Tear lipocalin-lipid complexes are known to reside in the aqueous,^21^^,^^37^^,^^38^ but none of the above referenced works addresses the localization of phospholipids at the surface of tears. Based on binding of phospholipids to tear lipocalin and published amounts, some have speculated that the available phospholipids in human tears would be insufficient to form a surfactant.^3^^,^^39^ Millar et al.^40^ and Mudgil and Millar^41^ studied the adsorption of holo- and apo-tear lipocalins in aqueous covered by human and bovine Meibomian lipids in Langmuir trough experiments. While both holo- and apo-tear lipocalin adsorbed, the surface pressure increased more with apo-tear lipocalin. Millar et al. concluded that phospholipids were not delivered to the surface when the complex was added to the subphase. However, meibomian films are not monolayers. Smaby and Brockman^42^ have demonstrated that phospholipids are known to be miscible with the types of complex lipids in tears and meibum. Surface pressure changes are sensitive to relative concentrations and packing arrays. The relatively small amount of miscible phospholipid in meibum might have a minimal effect on surface pressure. A further complicating factor is that tear lipocalin, like many other proteins, is known to unfold at the surface of water.^43^^–^^45^ Apo-tear lipocalin penetrates and rearranges at the surface faster than holo-tear lipocalin.^44^ This is related to the more flexible structure of the apo-form as lipid stabilizes the structure of holo-tear lipocalin.^46^^,^^47^ The possible existence of phospholipid on the surface of tears remains to be addressed.

The surface layer on human tears varies greatly in thickness (2.6–500 nm).^11^ The range of variability extends not only from subject to subject but also from day to day in the same subject and even in different areas of the same sample. Biochemical identification of components at the surface versus the bulk can be challenging. Mass spectrometry and Fourier transform infrared spectroscopy have successfully identified components in whole meibum and tears, but these techniques are not directly applicable to localization in the structure of thin films. However, recent advances in polarization-modulated Fourier transform infrared reflective absorption spectroscopy (PM-IRRAS) have garnered success in detection and study of orientation of surface components as thin as a monolayer.^48^^,^^49^ While many of the tear lipids have overlapping infrared spectral features, the phosphate group has relatively specific signals in the fingerprint region.^36^ PM-IRRAS has been shown to reveal spectral features of phospholipids in monolayers, including the fingerprint region.^48^ Therefore, PM-IRRAS in tandem with ellipsometry were the methods chosen to interrogate phospholipids using the tear film as a model.

## Methods

### Materials

Chemicals 1,2 dipalmitoyl-sn-glycero 3-phosphatidyl choline (DPPC) were purchased from Avanti Polar Lipids, Inc. (Alabaster, AL, USA), and sodium phosphate, oleic acid, and oleyl oleate were obtained from MilliporeSigma (St. Louis, MO, USA). Deionized water (17–18 megohms) was obtained from a Barnstead (Lake Balboa, CA, USA) 4 cartridge water purification system. Glass pipettes and vials were exclusively used in handling all lipids and solvents.

### Collection of Human Tears

Stimulated human tears were collected from 30 healthy volunteers (aged 18–23 years) in accordance with the tenets of the Declaration of Helsinki and approved by the institutional review board. Informed consent was obtained from donors after explanation of the possible consequences. Human participants were screened to exclude symptoms of dry eye disease as previously described.^50^ Stimulated tears were collected as previously published with polished glass tips and glass transfer pipettes.^33^ Tears were pooled into three lots each from 10 different participants that included men and women and placed in polytetrafluoroethylene-lined glass vials and stored under nitrogen at −80C° until use.

### PM-IRRAS

PM-IRRAS spectra were collected at 20°C to 25°C with a Michelson-style interferometer Nicolet i550 FT-IR spectrometer (Madison, WI, USA). Linearly polarized light was modulated at frequencies of 1.0 to 5.0 kHz corresponding to the wavenumber region of 800 to 4000 cm^−1^ using a photoelastic modulator, PEM-100, from Hinds Instruments (Hillsboro, OR, USA), which has an intrinsic resonance frequency of 50 kHz. The modulation frequency in a range of 1400 to 2000 cm^−1^ was varied for specific regions of the spectra. The alternately generated s- and p-polarized light traveled to a SPECAC (Orptinton, UK) monolayer grazing angle specular reflectance accessory, which was mounted on an aluminum breadboard purchased from Thorlabs (Newton, NJ, USA) and housed in a Plexiglas cabinet continuously purged with nitrogen gas. The angle of incidence was 82° relative to the optical axis normal to the surface interface of the sample. This angle was chosen to constrain and maximize the detected signal to surface components. The angle of incidence that optimizes the signal-to-noise ratio from surface films has been shown to be 71° to 90° for pure water, dependent on the optical geometry of the surface molecules.^51^ The reflected light from the sample was collected in a liquid nitrogen mercury cadmium telluride detector at 77°K with a spectral resolution of 4 cm^–^^1^. The PM-IRRAS signal was obtained with a synchronous sampling demodulator, SSD-100-15, from GWC Technologies (Madison, WI, USA). To increase the sensitivity, 5000 scans were collected and the signal averaged for each spectrum. The PM-IRRAS signal is a ratio spectrum, B/A, where B represents the differential spectrum between the polarization directions and A is the static sum, B/A ≈ C (J(φ) (R_p_ – R_s_))/(R_p_ + R_s_). C is a signal-processing constant. J(φ) is the second-order Bessel function of maximum dephasing. R_p_ and R_s_ are the intensities of polarized reflectivities of plane and perpendicularly polarized light.^51^ The phase shift for the s-polarized beam is close to 180°, irrespective of the angle of incidence or wave numbers, and essentially cancels. Therefore, the corresponding signal intensity for s-polarized light is very low as very little absorption occurs. The resulting surface selected spectra will show absorption mainly from the p-polarized beam. The surface selectivity was tested using sodium phosphate buffers, 0.1 and 1.0 M, with and without a monolayer of oleic acid. Spectra were normalized in OMNIC software from Thermo Fisher Scientific (Waltham, MA, USA) using the manufacturer's standard algorithm for linear baseline correction. This includes limiting the spectrum to the region of interest, manually joining points along the baseline while being careful not to introduce new peaks, and using a smoothing function if indicated. The resulting corrected spectra with B/A on the y-axis have a different form than typical IRRAS, generally devoid of negative signals.

### Ellipsometry

Imaging ellipsometry measurements were made with an imaging nulling ellipsometer nanofilm-EP4 from Accurion Gmbh (Göttingen, Germany) at 20°C. The ellipsometer was mounted on an active vibration isolator from Accurion Gmbh and housed in a customized cabinet to exclude air currents and stray light. Polarizer and analyzer angles were determined under the nulling conditions for each region of interest with a 5× objective. Thickness measurements were made in the nulling mode with a 658-nm, 50-mW laser light source as previously described.^11^ Measurements were collected at 0.5° intervals of incident angles between 50° and 57°. The data were modeled for a lipid surface layer under air and over an aqueous substrate (index of refraction, n = 1.333), all considered transparent films in the model. The refractive index of the surface tear film was also separately tested for fit using the stacked layered model.^52^ The optical constant n = 1.491 at a 658-nm wavelength matched that given for Meibomian lipids.^12^ The optical constants for oleic acid and DPPC were taken as n = 1.463 and 1.453, respectively.^9^^,^^53^ Angular measurements of the polarizer and analyzer reflect the polarization and phase shift and are referred to as psi (Ψ) and delta (Δ). Ψ and Δ values were measured at each pixel in the scanned area and averaged by the instrumentation software. The lateral resolution approximates 1 µm as given by the overall resolution provided by the optics in the reflected light beam. The reflectance (r) matrix is described by the ratio ρ = R_p_ /R_s_ = tan (Ψ) e^iΔ^, where the amplitude of the parallel (p) and orthogonal (s) components of the reflected light are normalized to initial amplitude of the incoming light with the ellipsometric angles Ψ and Δ, respectively. To obtain thickness, measured ellipsometric angles Ψ and Δ were parameters input into the Accurion proprietary modeling software (EP4Model software). The software uses a Berreman matrix algorithm for multilayered films fitting and a Levenberg-Marquardt multivariate regression algorithm to Fresnel equations for multilayered films.^52^^,^^54^ An adequate fit was taken as a root mean square error (RMSE) of less than 15, although for most fits, RMSE less than 5 was observed. The material stack included successive layers of air, lipid, and water. Lipids, gravimetrically prepared and dissolved in chloroform, were layered on the substrates by adding 1 µL to cover the surface area of the sample chamber as described.^11^ A minimum of six measurement scans were made from each sample in different regions that encompassed the thinnest and thickest measurements. Scans were performed in duplicate. The thicknesses are reported as means of the thicknesses measured at each pixel of the regions of interest in each sample. In some circumstances, image maps were obtained to visualize nonuniform regions. The maps were taken at an angle of incidence of 55° using two zones or settings for both the polarizer and compensator with two iterations at each pixel. The system was initially calibrated with a silicon dioxide wafer of known surface layer thickness.

### Sample Chamber

To measure the same samples for both PM-IRRAS and ellipsometry, a customized sample chamber was machined from black polytetrafluoroethylene. A rough bottom vitiated reflections from the chamber during ellipsometry. To obtain the maximum signal for PM-IRRAS, a relatively large chamber (70 × 23 mm) was needed (surface area = 1.61E17 Å^2^). The volume required to fill the chamber required pooled samples of tears rather than individual samples. Each sample was scanned successively on both the ellipsometer and the PM-IRRAS spectrometer.

## Results

### Evaluation of Surface Selection of PM-IRRAS

Experiments were performed with oleic acid monolayers to verify that mainly surface molecules can detected by the PM-IRRAS instrument as configured. Thicknesses measured with ellipsometry confirmed the presence of a monolayer (Table 2) and were consistent with previously published ranges.^11^ Tear film thicknesses were predictably variable due to the irregular surface properties that have been well documented.^11^^,^^55^

**Table 2.** Surface Thicknesses Measured by Ellipsometry

Sample	Thickness, Mean ± SD, nm
Water	0.0 ± 0.1
Sodium phosphate	0.3 ± .2
Oleic acid monolayer	1.3 ± 0
DPPC monolayer	2.5 ± .2
Oleic acid monolayer/sodium phosphate	1.9 ± .3
Tears (lot 1)	7.0 ± .1
Tears (lot 2)	7.7 ± .1
Tears (lot 3)	3.0 ± .1
Tears (lot 2) + 5 nm DPPC	2.1 ± .1
Tears (lot 3) + 5 nm oleyl oleate	5.8 ± .1

**Figure 2.**
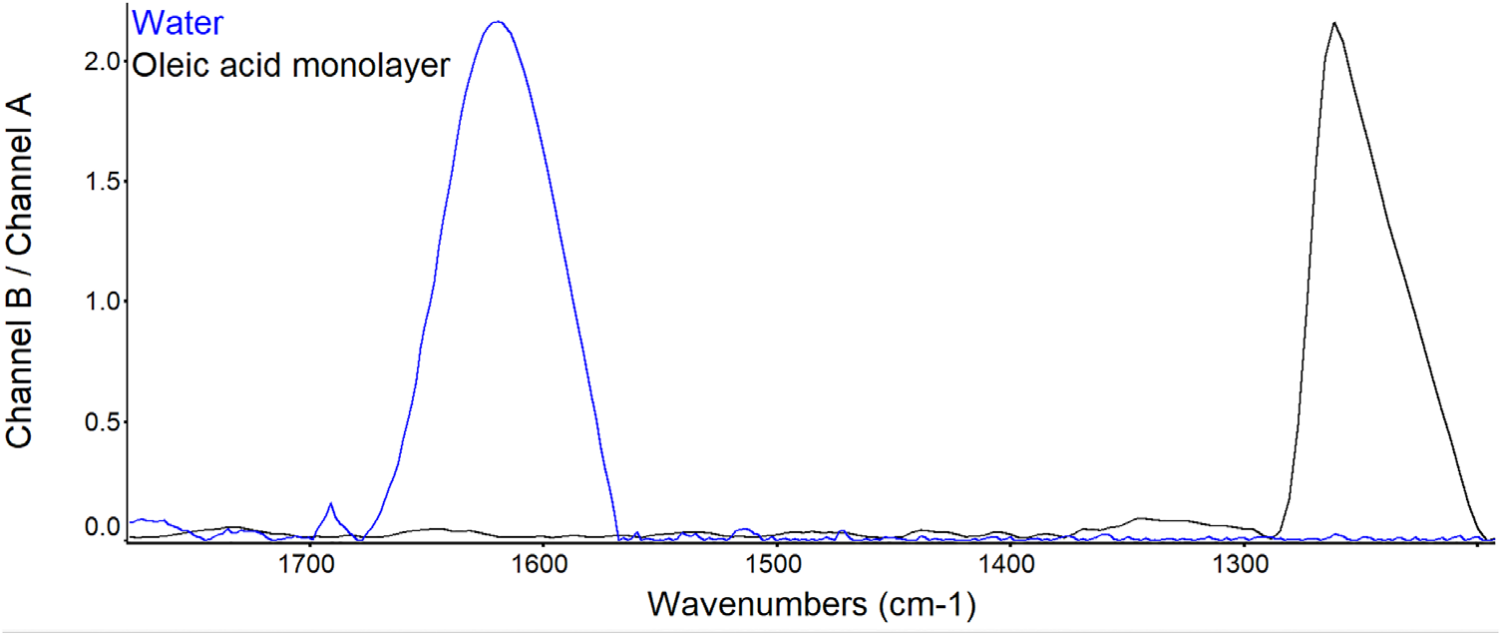
PM-IRRAS spectra before and after an oleic acid monolayer film of 1.3 nm thickness was created on water. The spectra were averaged over 5000 scans. The band at 1660 cm^−1^ is assigned to OH stretch; the band at 1260 cm^−1^ is assigned to the alkyl groups of oleic acid. Full scale for each sample is shown. The y-axis shows the scale for water.

PM-IRRAS spectra of water show bands in several regions of the infrared spectrum. For example, there is a strong absorption peak assigned to OH stretch at 1290 cm^−1^. A monolayer of oleic acid 1.3 nm thick appeared to effectively suppress the water absorption signal at 1660 cm^−1^ (Fig. 2). The peak at 1255 cm^−1^ can be associated with methyl groups of oleic acid. Interestingly, a peak at 1750 cm^−1^, carbonyl stretch, was minimal in the monolayer, although it could be seen in the PM-IRRAS spectra of pure oleic acid.

**Figure 3.**
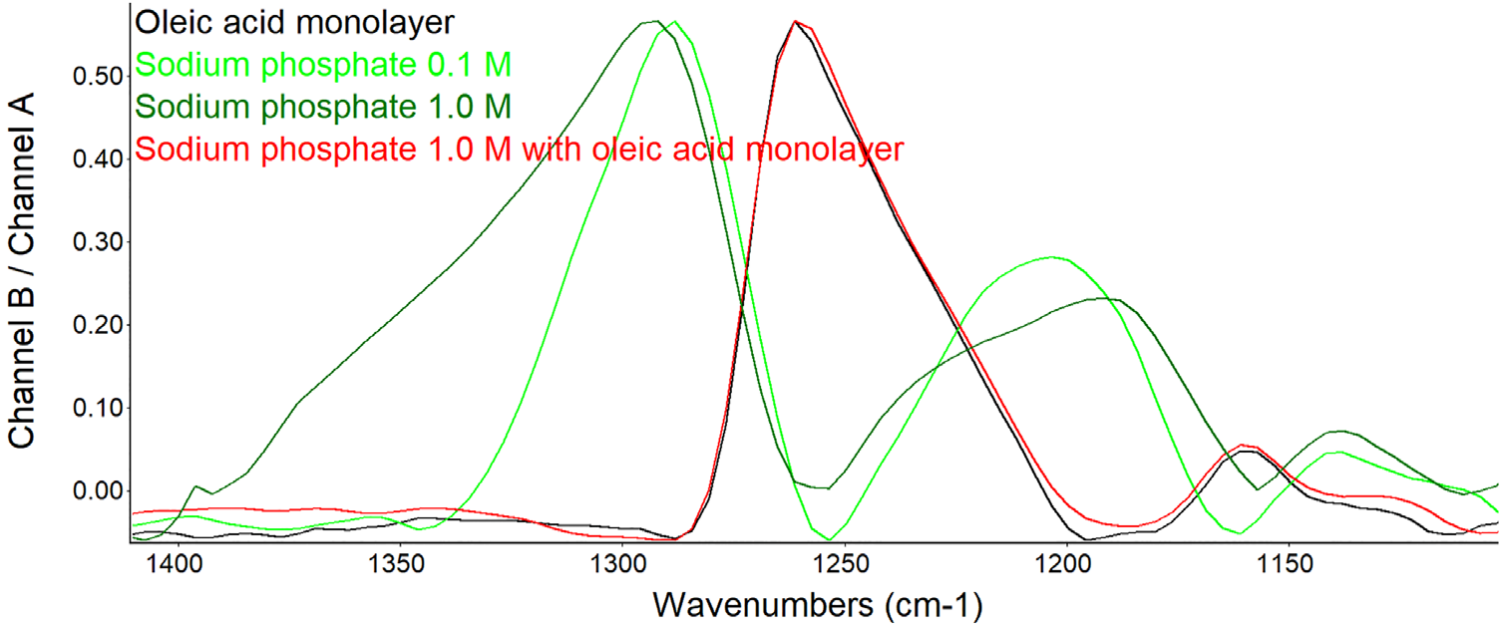
PM-IRRAS spectra before and after an oleic acid monolayer film was created over sodium phosphate buffer. The spectra were averaged over 5000 scans. The broad bands at 1300, 1175, and 1140 cm^−1^ associated with sodium phosphate buffer are suppressed by oleic acid. The band at 1260 cm^−1^ is assigned to the alkyl groups of oleic acid. Shown at full scale for each sample to identify relative peak positions. The y-axis scale to the *left* is shown for that of 1.0 M sodium phosphate.

To determine if signals derived from the phosphate group could be detected under conditions of PM-IRRAS, buffers containing sodium phosphate were investigated. The spectra revealed characteristic phosphate absorption signals in the fingerprint region of the infrared spectrum (Fig. 3). Peaks at 1180 to 1200 cm^−1^ are known to be associated with the phosphate group and assigned to P = 0 stretch. The absorption signals were increased in height, broadened, and shifted slightly at the higher concentration of sodium phosphate as expected. These phosphate signals appeared suppressed by a monolayer of oleic acid (Fig. 3).

### Phospholipid on the Surface of the Human Tear Film

**Figure 4.**
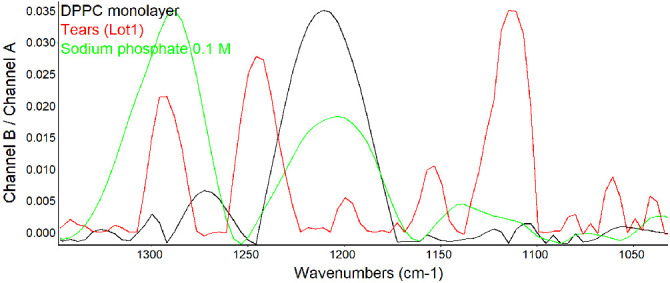
PM-IRRAS spectra in the fingerprint region for phosphate signals at the surface of human tears shown at full scale for each sample. The y-axis shows the scale for tears.

In exploring the possibility that phosphate signals from phospholipid monolayers were also detectable, DPPC was added to the surface of water (Table 2 and Fig. 4). The bands observed on the DPPC monolayer were of low intensity but detectable and overlapped the bands detected with sodium phosphate. Pooled human tears were also investigated and showed even weaker but definite signals in the same region (Fig. 4). The bands at about 1290, 1200, and 1040 cm^−1^ directly overlapped the bands of both DPPC and sodium phosphate with minor shifts.

**Figure 5.**
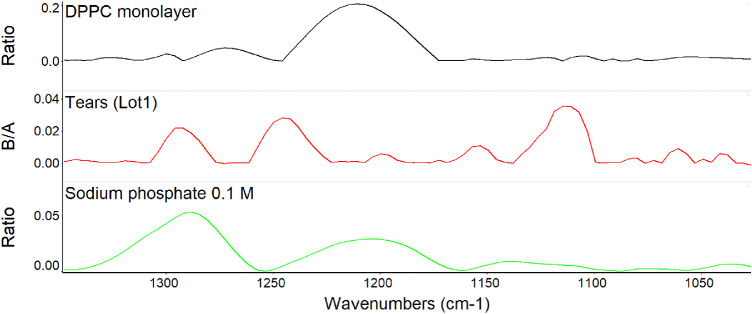
Stacked PM-IRRAS spectra from Figure 4 to show the relative signal intensity of each sample.

Comparison of the scales in Figures 3 and 4 reveals a decreased intensity of the phosphate signal in tears by approximately an order of magnitude versus 1.0 M sodium phosphate buffer. Stacked spectra for each analyte in Figure 4 allow for direct comparison of the scales of tears, DPPC monolayer, and 0.1 M sodium phosphate buffer (Fig. 5). The distribution of signal intensity varies for each sample with low-intensity signals from tears but close to that of 0.1 M sodium phosphate.

**Figure 6.**
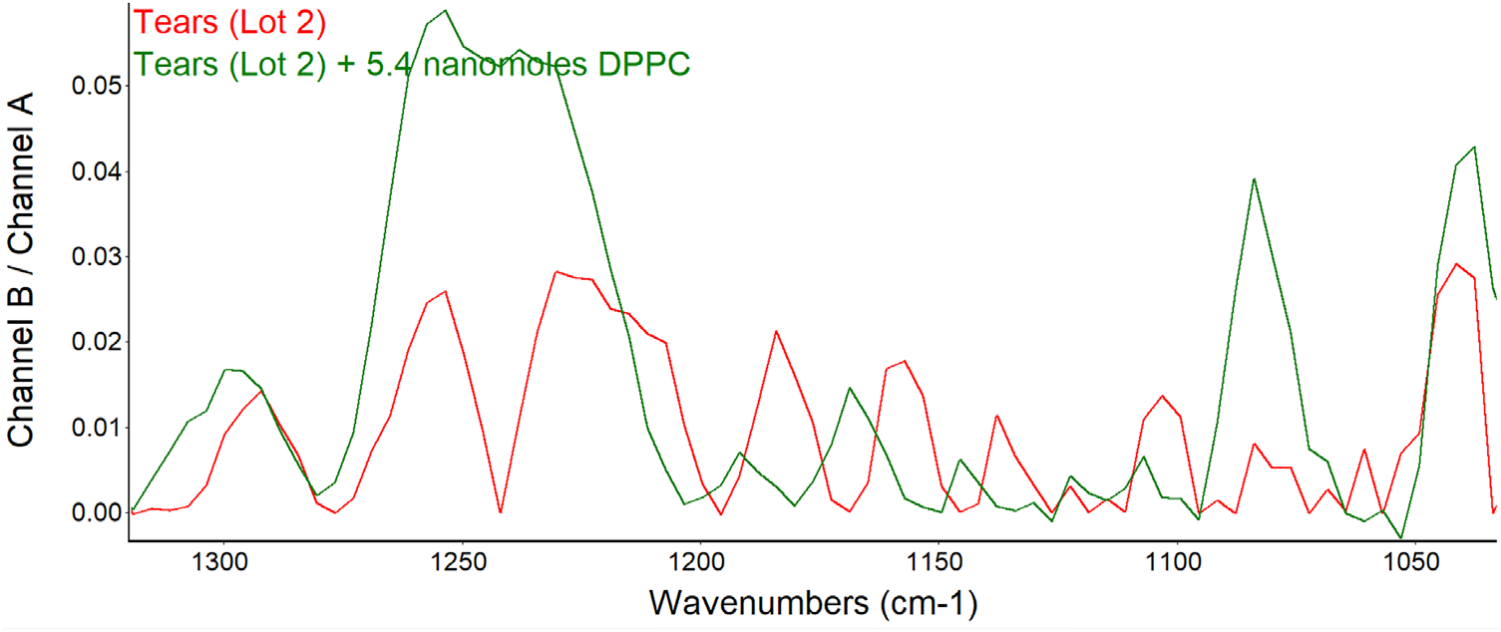
PM-IRRAS spectra in the region of phospholipid signal before and after the addition of 5 nmol DPPC to tears. For comparison, the B/A ratio is shown as the common scale (applies to both samples).

To further verify that the signal pattern observed was due to phospholipids, DPPC was added to another lot (2) of human tears. The signal intensity broadened and increased at about 1200 to 1290 cm^−1^, and peaks remained at 1160 to 1200 cm^−1^ and were augmented at 1070 and 1040 cm^−1^ (Fig. 6).

**Figure 7.**
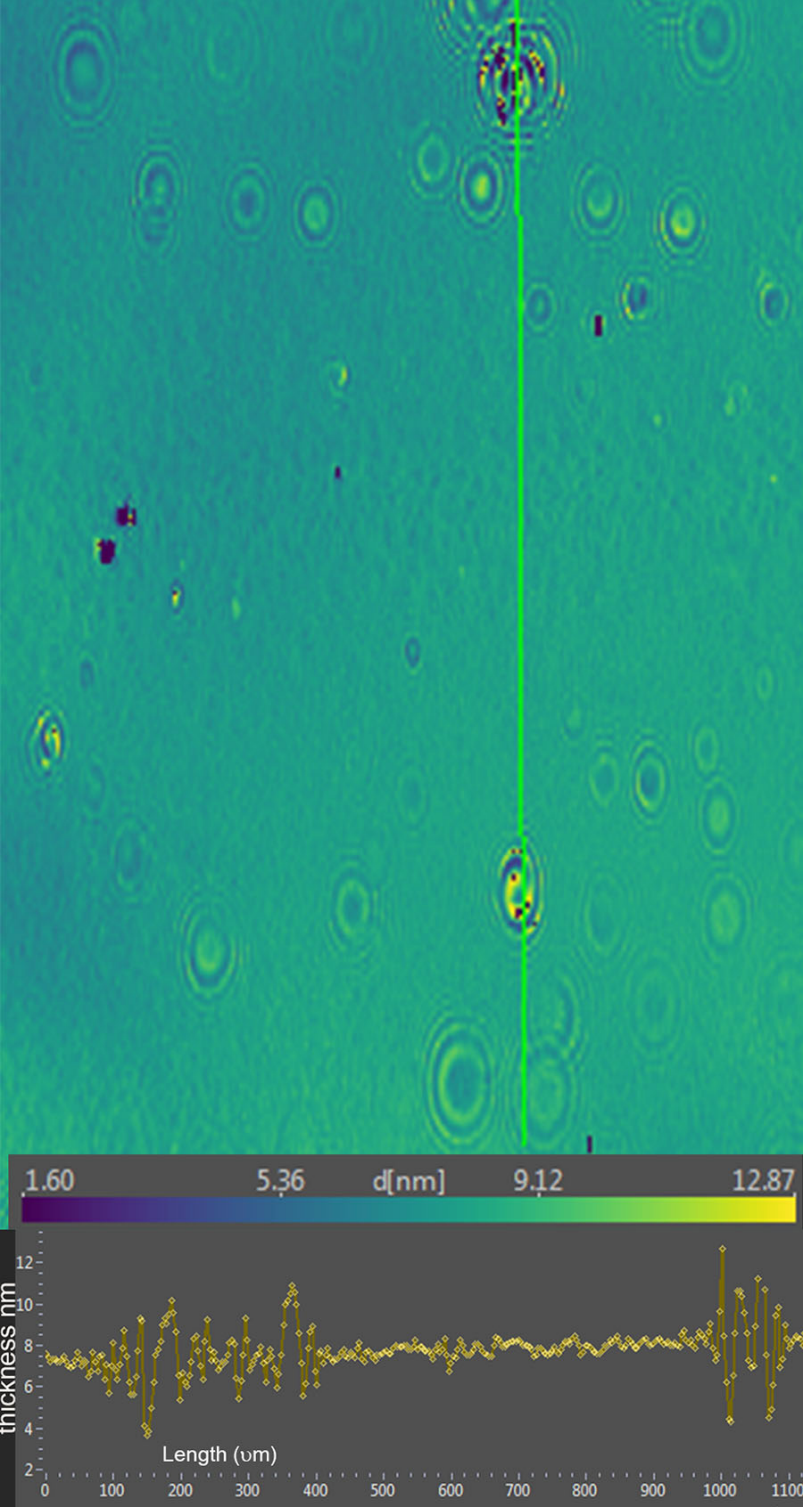
*Upper portion*, ellipsometric map of human tears (lot 2) prior to addition of phospholipid. Thickness at each pixel, indicated as d [nm], is color coded. The x- and y-axis scales are shown in µm. *Bottom*, thickness profile for the *green line* in the map.

Ellipsometric imaging of the tears typically showed variation in thickness with apparent islands raised above the surface (Fig. 7, Table 2). In lot 2, the peaks measured up to about 12 nm (Fig. 7). The addition of 5 nm DPPC coincided with a decrease in the average thickness of the surface film by ellipsometry (Table 2).

**Figure 8.**
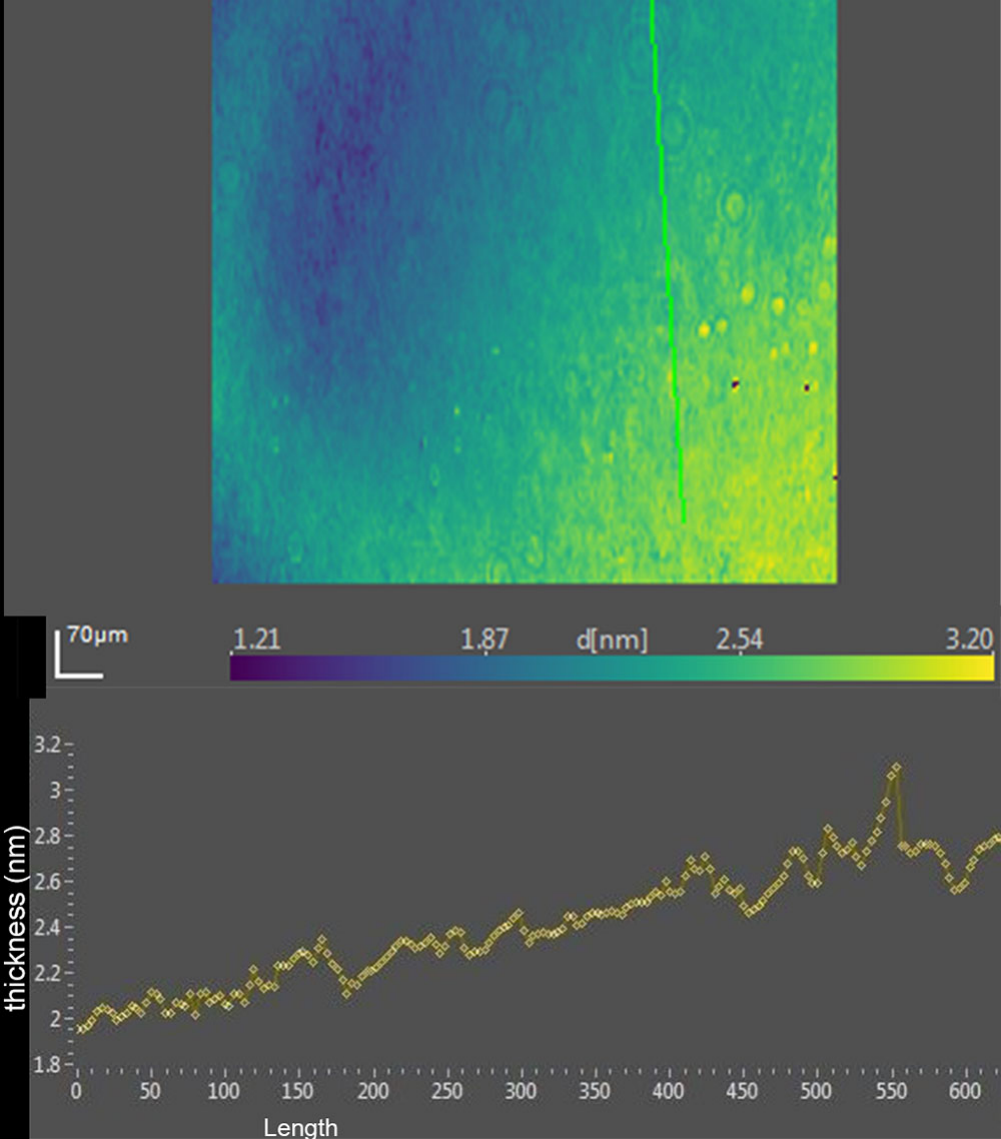
*Upper*, ellipsometric image map of human tears (lot 2) after the addition of 5.4 µmol DPPC. Thickness at each pixel, indicated as d [nm], is color coded. The x- and y-axis scales are shown in µm. *Bottom*, thickness profile corresponds to the *green line* in the map.

Ellipsometric imaging was also performed of the human tear film (lot 2) after the addition of DPPC. The surface islands seen with tears alone were replaced with a more uniform but thinner profile (Fig. 8).

**Figure 9.**
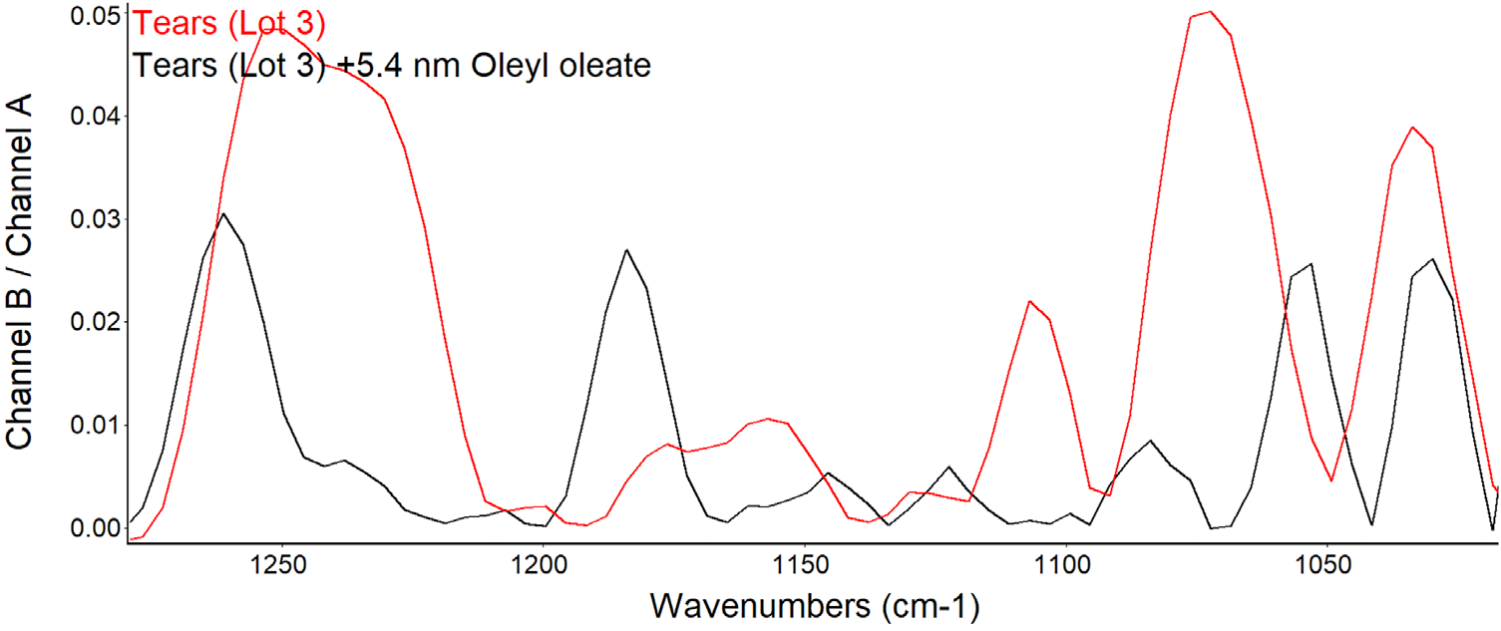
PM-IRRAS spectra in the region of phospholipid signals before and after the addition of 5.4 nmol oleyl oleate to the surface of tears. For comparison, the B/A ratio is shown as the common scale (applies to both samples).

Tears and meibum contain abundant wax esters. Because wax esters absorb between 1100 and 1300 cm^−1^, spectra of tears before and after the addition of oleyl oleate were compared (Fig. 9). Oleyl oleate was chosen as the wax ester because the lipid is a liquid at room temperature, which facilitates spreading on the surface of tears.

**Figure 10.**
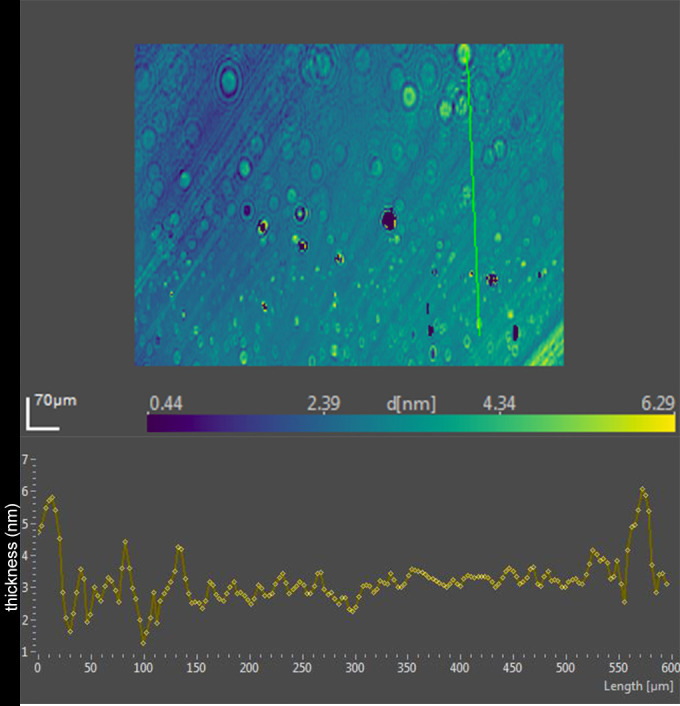
*Upper*, ellipsometric image map of human tears (lot 3) prior to the addition of oleyl oleate. Thickness at each pixel, indicated as d [nm], is color coded. The x- and y-axis scales are shown in µm. *Bottom*, thickness profile corresponds to the *green line* in the map.

The bands in the phosphate region for lot 3 of tears were similar to previous lots. The addition of oleyl oleate resulted in a reduced peak height at 1200 to 1272, 1067, and 1043 cm^−1^. Increased signals were evident at 1180 and 1122 cm^−1^. Ellipsometric data for this lot of tears showed less average thickness than prior lots (Table 2). The ellipsometric map of this lot of tears was similar to that of lot 2 with raised discrete regions that contributed very little to the average thickness (Fig. 10, Table 2).

**Figure 11.**
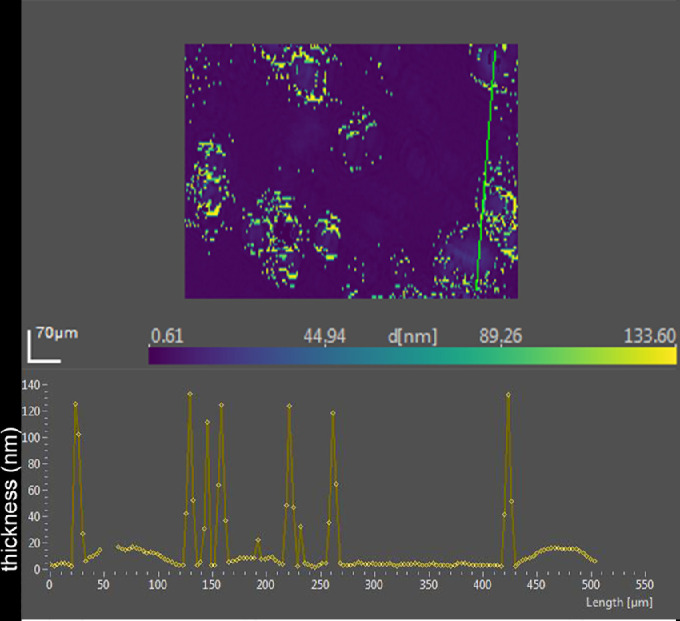
*Upper*, ellipsometric image map of human tears (lot 3) after the addition of 5.4 nmol oleyl oleate. Thicknesses at each pixel, indicated as d [nm], are color coded. The x- and y-axis scales are shown in µm. *Bottom*, thickness profile corresponding to the *green line* in the map.

A distinct pattern change emerged upon the addition oleyl oleate to tears (Figs. 10 and 11). Although enough oleyl oleate was added to form a monolayer, the pattern changed to a slightly thicker average tear film attributable to apparent domains composed of very steep rimmed islands measuring over 100 nm in thickness (Fig. 11). Inside the rims, raised mounds of about 10 to 20 nm were observed (Fig. 11).

## Discussion

The two major findings of this study are (1) signals obtained from PM-IRRAS performed under grazing conditions can be limited to signals from only the surface monolayer rather than the subphase, and (2) signals so obtained localized phospholipids at the tear film surface.

In PM-IRRAS, lipid monolayers have been demonstrated to suppress the signal from carbonate ions in the subphase.^51^ Our data are consistent with suppression of the large and broad OH– signal from water and those assigned to phosphate in buffer by a monolayer of oleic acid (Figs. 2 and 3). Given the expected phospholipid bound to tear lipocalin in the subphase, this result was a prerequisite to interrogate the presence of the phospholipids at the surface of tears. Ellipsometry of the sodium phosphate buffer revealed that the average thickness did not fit perfectly to the model of a surface layer of n = 1.463, and mapping revealed clusters. The clusters may represent aggregates in the buffer. This could account for the slightly increased thickness of the oleic acid monolayer on the surface of sodium phosphate buffer compared to that over water (Table 2).

The finding of consistent PM-IRRAS bands that can be assigned to phospholipids in three lots of tears provides compelling evidence that the surface of tears contains phospholipids. The bands with peaks at about 1272, 1067, and 1043 cm^−1^ match those assigned by Borchman et al.^36^ to phospholipids. Although not perfectly overlapping, these signals appear augmented by addition of DPPC to tears to further support the assignment to phospholipids (Fig. 5). Minor differences may be due to inevitable sample variation and shifts that can be seen with PM-IRRAS compared to transmission FTIR as well as interactions of phospholipids with other tear components. The phosphate signal intensity in tears is similar to that of 0.1 M sodium phosphate. The concentration of phospholipid bound to tear lipocalin (Table 1) in the subphase is three orders of magnitude less than phosphate ion present in 0.1 M sodium phosphate, effectively excluding the subphase as a source of the signal. The phosphate signal in tears is more than an order of magnitude less than that of the DPPC monolayer (Fig. 5). PM-IRRAS is predicated on the principle that s-polarized light gives no surface electric field and no surface absorption, and polarization modulation is efficient to discriminate between near-surface absorbance versus from isotropic strong absorption deeper in the sample. The reduction in amplitude of the B/A ratio almost certainly reflects a lower concentration of phospholipid at the surface of tears than in the monolayer of DPPC. Considering the other lipids that have been extracted from tears (Table 1), the phospholipids at the surface may be diluted with other surface active components such as those in Table 1, as well as more minor components such as o-acyl-w-hydroxy fatty acids. The ellipsometry maps suggest that the DPPC added to the tear film displaced other surface components in tears accounting for the smoothing and thinning (from 7 nm to 2.6 nm; Table 2) of the surface layer. Displacement may have been facilitated by the enhanced spreading of DPPC over the mixture of lipids in tears. The total amount of lipids probably exceeded that needed for a monolayer. Also, the tendency for some lipids to bind polytetrafluoroethylene may have enhanced displacement.^56^ Although wax esters may show some absorption in the 1100- to 1300-cm^−1^ region, addition of oleyl oleate to tears revealed no increase in the peak heights previously assigned to phospholipid. This suggests the peak assignments are rational and phospholipid remained in the mixture.

### Implications for Tear Film Surface Structure

The maps of lots of pooled tears represent an ensemble average of many participants. Notwithstanding a great variation in pattern and thickness of the surface layer of tears among individual samples and regions of the same sample, the maps show similar islands.^11^^,^^55^ The current data may shed some insight into the nature of the islands in tears. The reduction in average thickness of the surface film of tears by addition of DPPC is consistent with the mixing and finally displacement of wax and/or cholesteryl esters by more surface-active phospholipids. The formation of very thick rims around the perimeter of these raised islands by addition of wax ester suggests the lipids aggregate in thicker masses (Figs. 10 and 11). The thinning between these micro-volcano-like islands suggests other components may have joined the aggregates with film collapse. Phospholipid bands are still observed from the surface. These findings are consistent with the ability of phospholipids to mix with other surface-active lipids, including wax esters and cholesteryl esters, to form rapidly spreading surface films.^57^^,^^58^ Phospholipids are known to be miscible with other lipids, including cholesteryl esters, triglycerides, fatty alcohols, and fatty acids.^42^^,^^59^ The findings are consistent with model tear lipid systems that show phospholipids are located between neutral lipids.^4^ Paananen et al.^60^ emphasized the importance of wax esters in gel formation in tears to prevent evaporation. The studies here fit the hypothesis that a complex arrangement of lipids exists on the tear film that includes phospholipids at the surface. The ratio of components may be critical as a relatively large amount of phospholipid may obviate ideal packing and cause displacement of wax esters.^42^ Likewise a high ratio of wax esters results in slower-spreading surface films and could promote aggregation and destabilize the tear film.^57^ Further, the displacement of thick aggregates by DPPC fits data on the published effect of DPPC on Meibomian aggregates in surface film studies.^61^ Increased reciprocal compressibility and surface pressures suggest phospholipids would have a favorable functional role to stabilize the tear film. However, direct evidence that the raised areas observed in tears are wax or cholesteryl esters is lacking in the data here. The extensive overlap of chemical functional groups (e.g., alkyl chains, carboxyl groups) and complexity of tear components (Table 1) vitiates unambiguous assignment by PM-IRRAS.

### Role of Phospholipid Binding Proteins in Tears

Most phospholipids in tears are bound to tear lipocalin; the complex has been assumed to reside exclusively in the bulk of aqueous.^39^ The data herein challenge that assumption and provide strong evidence to localize some phospholipid at the surface of the tear film. While the lipocalin-phospholipid complex may be mainly present in the aqueous, one must consider the obligatory chemical equilibrium between phospholipid bound to tear lipocalin and that unbound in aqueous as dictated by the dissociation constant. The dissociation constants for tear lipocalin-phospholipid complexes, including various analogs, have been measured in the range from 0.15 to 1.5 µM.^38^^,^^62^ The concentrations of both tear lipocalin and phospholipids in tears are known (Table 2). For the purpose of calculation, one can assume the vast majority of the phospholipid is bound to tear lipocalin and use the more conservative dissociation constant. In this case, the concentration of tear lipocalin-phospholipid complex (TL-P) effectively equals the concentration of total phospholipid (30 µM). The concentration of free tear lipocalin (TL is the total concentration, 74 µM, minus that bound [30 µM]) is 44 µM. The equation for dissociation can be solved for unbound phospholipid (P), P = [Kd] [TL-P] / TL or P = [.15] [34 µM / [44 µM] = 0.1 µM. Campbell et al.^26^ estimated that the molecular area of phospholipid is 85 Å^2^, the area of the ocular surface is 3.5 × 10^7^ Å^2^, and the average tear volume is about 6.5 µL. This equates to about 411,765 molecules of phospholipid required to form a monolayer on the ocular surface. The concentration of calculated unbound phospholipid molecules at equilibrium in tears, 0.1 µM, is about 6 orders of magnitude more phospholipid than needed to form a monolayer. This is a conservative estimate since only simple dissociation of phospholipid in aqueous is considered. Zwitterionic phospholipids in tears fit Tanford's principle of opposing forces that result in low free energy in stabilization of surface films.^63^ A position at the tear-air interface for unbound phospholipid is therefore energetically favored over residence in the bulk. Another factor that may promote adsorption of phospholipid to the surface tear film is phospholipid transfer protein. Although present in very small amounts in tears, phospholipid transfer protein has been shown to transfer phospholipids from vesicles to unlabeled HDL acceptors.^64^ It is plausible that phospholipid transfer protein could transfer free phospholipid residing in the subphase to the air interface of tears.

While the amount of phospholipid is more than adequate to form a monolayer, the surface of the tear film rarely manifests as just a monolayer.^11^^,^^55^ The major surface lipids on the tear film, wax and cholesteryl esters, have been shown to mix with phospholipids.^42^ One must also consider the possibility that a mixture of lipids and proteins, specifically the complex of phospholipid and tear lipocalin, is adsorbed at the surface. Millar's group provided evidence for such a possibility in Langmuir trough experiments.^40^^,^^41^^,^^65^ PM-IRRAS is unable to unambiguously identify lipocalin at the surface of tears because of the complex nature of tears. Many proteins and lipids in tears feature bands where amide signals are seen, 1650 cm^−1^. One cannot exclude phospholipid bound to protein at the surface of tears from this study.

### Implications for Dry Eye Disease

The finding that excess phospholipid may replace other lipids on the tear film may have implications for lipids incorporated in artificial tears solutions. Several products on the market contain phospholipids with varying effectiveness. Adjusting the composition to reflect a product with a specific mixture of lipids may be an important strategy to form a stable tear film.

### Challenges and Limitations of This Study

The assignment of phospholipid in the PM-IRRAS spectra of tears extends the work of Borchman et al.,^36^ who interrogated the fingerprint region in transmission infrared spectra to identify these lipids in tear samples with only 50 scans. Thousands of scans were necessary to provide an adequate signal for PM-IRRAS in this study. However, the phosphate signals detected are clearly not an artifact because the signal amplitudes match the concentration in buffers and those expected from monolayers of the lipids. The analysis of complex mixtures is limited because other lipids contribute to the absorption in the fingerprint region. Another constraint is the relatively large volume needed for PM-IRRAS studies, which necessitated the pooling of individual tear samples in this study. The variation in thickness and patterns of the lipid layers of tears of individual subjects may not be well represented. It is somewhat reassuring that the data were reasonably similar from all three pooled lots of tears, especially when considering the variation between subjects in prior imaging studies.^11^^,^^55^

PM-IRRAS can be used to identify lipids in complex biologic surface films. The data from PM-IRRAS, in tandem with ellipsometry, provide evidence that phospholipids are present at the tear film surface. This conclusion seems reasonable from calculations predicted by equilibrium binding to tear lipocalin as well as the prominent surface activity of phospholipids. Imaging ellipsometry monitors thickness and pattern changes in biologic films and is amenable to probing the films with addition of lipids. In this instance, wax esters interact with other lipids at the surface and alter the topography of tears. The techniques provide insight into a tear film structure where phospholipids reside at the surface and interact with other tear lipids.
